# Lipid nanoparticles-loaded with toxin mRNA represents a new strategy for the treatment of solid tumors

**DOI:** 10.7150/thno.82228

**Published:** 2023-06-12

**Authors:** Yasmin Granot-Matok, Assaf Ezra, Srinivas Ramishetti, Preeti Sharma, Gonna Somu Naidu, Itai Benhar, Dan Peer

**Affiliations:** 1 Laboratory of Precision NanoMedicine, Shmunis School for Biomedicine and Cancer Research, George S. Wise Faculty of Life Sciences, Tel Aviv University, Tel Aviv 69978, Israel.; 2 Center for Nanoscience and Nanotechnology, Tel Aviv University, Tel Aviv 69978, Israel.; 3 Department of Materials Sciences and Engineering, Iby and Aladar Fleischman Faculty of Engineering, Tel Aviv University, Tel Aviv 69978, Israel.; 4 Cancer Biology Research Center, Tel Aviv University, Tel Aviv 69978, Israel.; 5 Laboratory of Antibody Engineering, Shmunis School for Biomedicine and Cancer Research, George S. Wise Faculty of Life Sciences, Tel Aviv University, Tel Aviv 69978, Israel.

**Keywords:** lipid nanoparticles, mRNA, cancer therapy, gene therapy, suicide-gene therapy, immunotoxins

## Abstract

**Background and rationale**: Cancer therapy have evolved remarkably over the past decade, providing new strategies to inhibit cancer cell growth using immune modulation, with or without gene therapy. Specifically, suicide gene therapies and immunotoxins have been investigated for the treatment of tumors by direct cancer cell cytotoxicity. Recent advances in mRNA delivery also demonstrated the potential of mRNA-based vaccines and immune-modulators for cancer therapeutics by utilizing nanocarriers for mRNA delivery.

**Methods:** We designed a bacterial toxin-encoding modified mRNA, delivered by lipid nanoparticles into a B16-melanoma mouse model.

**Results**: We showed that local administration of LNPs entrapping a modified mRNA that encodes for a bacterial toxin, induced significant anti-tumor effects and improved overall survival of treated mice.

**Conclusions:** We propose mmRNA-loaded LNPs as a new class of anti-tumoral, toxin-based therapy.

## Introduction

mRNA therapeutics have gained great interest in the last years, being developed to treat a wide range of diseases including rare genetic diseases, neurodegenerative diseases, inflammation, cancer and infectious diseases. Like DNA molecules, mRNA molecules have the potential to express any protein of interest in diseased cells, using the cell's own protein synthesis machinery 1-4.

Unlike DNA, mRNA lacks the risk of insertional mutagenesis since nucleus entrance is not required for its functionality. However, mRNA is less stable than DNA and susceptible to both extracellular and intracellular degradation, caused by either unspecific RNase activity or immunogenicity triggered by Toll-like receptors mediated RNA recognition 1,4-7. Because of such limitations, DNA was preferred over mRNA for gene therapy development until recent years 8-13. Specifically, viral vectors and naked or plasmid DNA were mostly investigated for suicide gene therapy purposes, using intracellular expression of a toxic protein or an enzyme that converts a non-toxic compound to a cytotoxic molecule to induce cancer cell death 8,14-19.

Another biological class of drugs aiming to induce tumor cell death are recombinant or conjugated immunotoxins, which are antibody-toxin chimeric proteins 20-24. Moxetumomab pasudotox (Lumoxiti), for instance, is an anti-CD22 Fv murine antibody fused to PE38, a 38 kDa truncated form of pseudomonas exotoxin A (PE) that was approved by the U.S. Food and Drug Administration for the treatment of relapsed or refractory hairy cell leukemia 25-27. The PE domain used for immunotoxins is a NAD^+^-diphthamide ADP-ribosyltransferase that targets and inactivates eukaryotic translation elongation factor 2 (eEF2), leading to apoptosis 24,28-32. The conjugation of this domain to a specific antibody against cancer-related receptor, is intended to enhance drug specificity and lower the risk of adverse effects 20,31,33.

Although those immunotoxins have reached the clinic, they have been shown to have inherent characteristics causing resistance 33. Their mechanism of action includes target receptor binding, internalization, intracellular toxin processing and trafficking 22,27,30,32-33. All these processes are prone to resistance such as decreased cell-surface antigen presentation, impaired processing of the toxin or toxin cleavage in the lysosome 33.

To overcome some of the anti-immunotoxins resistance mechanisms, while avoiding potential risks of DNA-based suicide gene therapy, we suggest an mRNA-based therapy as an alternative. Herein, we used modified mRNA encoding the pseudomonas exotoxin A domain, a toxic domain originally produced by the bacteria pseudomonas aeruginosa. We entrapped *in-vitro* transcribed modified mRNA (mmRNA) encoding for this toxin in lipid nanoparticles (LNPs), and intratumorally administered them to B16-melanoma tumor-bearing mice. We hypothesize that such platform represents an improved approach for toxin-based anti-tumor therapy, with better safety profile and low immunogenicity while maintaining high potency.

## Methods

### Cell culture growth and maintenance

Monolayers of B16F10.9 (murine skin melanoma) cells were grown in T-75 flasks. The cells were cultured in Dulbecco's modified Eagle's medium (DMEM) containing 10% fetal bovine serum, penicillin (100 U per mL), streptomycin (0.1 mg/mL), nystatin (12.5 U per mL) and L-glutamine (2 mM). All cells were routinely checked every 2 months for mycoplasma contamination using the EZ-PCR Mycoplasma Test Kit (Biological Industries, Israel).

### LNP preparation

DLin-MC3-DMA (MC3) and EA-PIP were synthesized according to a previously described method 34-35. Firefly Luciferase, EGFP and PE mmRNA (custom- synthesized, sequence shown in supplementary data) modified mRNA were purchased from TriLink Biotechnologies, having a 5'-methoxy uridine modification and a Cap1 structure. Cholesterol, DSPC (1,2- distearoyl-sn-glycero-3-phosphocholine), polyethylene glycol (PEG)- DMG (1,2-dimyristoyl-rac-glycerol), and DSPE (1,2-distearoyl-snglycero-3-phosphoethanolamine)-PEG were purchased from Avanti Polar Lipids Inc. Briefly, one volume of lipid mixture (ionizable lipid, DSPC, cholesterol, DMG-PEG, and DSPE-PEG at 50:10.5:38:1.4:0.1 molar ratio) in ethanol and three volumes of designated mmRNA (1:10 molar ratio RNA to ionizable lipid) in a citrate buffer, pH 4.5 were injected into a NanoAssemblr microfluidic mixing device (Precision Nanosystems Inc.) at a combined flow rate of 12 mL min^-1^. The formed LNPs were dialyzed twice against PBS (pH 7.4) for 16 h to remove ethanol.

### Size distribution

mmRNA-LNPs size distribution and ζ potential were determined by dynamic light scattering using a Malvern Nano ZS ζ sizer (Malvern Instruments). For size measurements, LNPs were diluted 1:20 in PBS. For ζ potential measurements, LNPs were diluted 1:200 in double distilled water.

### Transmission electron microscopy

A drop of an aqueous solution containing LNPs was placed on a carbon-coated copper grid, air dried and analyzed using a JEOL 1200 EX transmission electron microscope.

### LNP quantification and encapsulation

To quantify the RNA in LNPs and to determine the RNA encapsulation efficiency, the Quant-iT RiboGreen RNA assay (Life Technologies) was used as previously described 36. Briefly, 2 µL of LNPs or dilutions of ribosomal RNA at known concentrations were diluted in a final volume of 100 µL of TE buffer (10 mM tris-HCl, pH 7.4 and 20 mM EDTA) in the presence or absence of 0.5% Triton X-100 (SigmaAldrich) in a 96-well fluorescence plate (Costar, Corning). The plate was incubated for 10 min at 40°C to allow particles to become permeabilized before adding 99 µL of TE buffer and 1 µL of RiboGreen reagent to each well. Plates were shaken at room temperature for 5 min, and fluorescence (excitation wavelength of 485 nm and emission was measured using a plate reader (BioTek Industries) according to the manufacturer's protocol.

### LNP transfection

Cells were counted using trypan blue (Biological Industries), and 10^5^ cells/well were placed in 24-well tissue culture plates (Greiner Bio-One, Germany) with 0.5 mL of growing medium, or 10^4^ cells/well were placed in 96-well tissue culture plates (Greiner Bio-One, Germany) with 100 µl growing medium. MC3 or EA-PIP LNPs were added to the wells at RNA amounts of 0.06 to 1 mg/ mL. Cells were incubated with the LNPs under standard culture conditions for up to 48 h. Then, cells were washed three times, incubated in fresh culture medium, and were taken for functional Luciferase assay system (Promega), XTT cell proliferation assay (Biological Industries) or FACS analysis for EGFP expression or stained with APC Annexin V (BioLegend) and propidium iodide (SigmaAldrich) according to the manufucturers' recommendations and then analyzed by FACS (Cytoflex, Beckman Coulter, USA. 2X10^5^ cells per FACS measurement).

### B16F10.9 tumor-bearing mice

Eight to thirteen weeks old female C57BL/6 mice (Envigo, Rehovot, Israel) were injected subcutaneously to the right flank with 10^5^ B16F10.9 cells suspended in 50 µL HBSS. For *in-vivo* imaging experiments, we used B16F10.9 cells stably transduced by a lentivirus to express mCherry and Firefly luciferase reporter proteins. Treatment start point was determined when the tumor volumes reached 40 to 50 mm^3^, or for imaging experiments, when labeled tumors reached luminescence of at least 4×10^7^ p/s, 8-12 days post tumor inoculation. The tumor dimensions were measured using Caliper, and tumor volume was calculated as: (width)^2^×length/2. For imaging experiments, we imaged mice using the *in-vivo* imaging system (IVIS, PerkinElmer). To observe luminescence, we intraperitoneally injected mice with 200 µL of 0.15 mg/mL XenoLight D-Luciferin Potassium Salt (PerkinElmer) reconstituted in PBS, and the mice were imaged 5 min post injection. The mice were randomly separated into three groups (n = 6 per group): PBS, mmFluc and mmPE. mmLNPs were intratumorally injected at a dose of 0.15 mg/Kg every 2-3 days.

### Histology

All histological sectioning, staining and histological evaluation were done by Dr. Zohar Gavish, Patho-Logica, Ness-Ziona, Israel. Histological evaluation and scoring were performed by Dr. Emmanuel Loeb, a Veterinary Pathologist.

### Animal experiments

All animal protocols were approved by the Tel Aviv University Institutional Animal Care and Usage Committee and in accordance with current regulations and standards of the Israel Ministry of Health. Mice were randomly divided at the beginning of each experiment.

### *In-vivo* biodistribution study and expression kinetics

Eight to ten weeks old female C57BL/6 mice (Envigo, Rehovot, Israel) were inoculated subcutaneously to the right flank with 10^5^ B16F10.9 cells suspended in 50 µL HBSS. 10 days post inoculation, mice were intratumorally (I.T.) injected with mmFluc-LNPs (mRNA dose: 0.15 mg/Kg). At 6 h post-injection, mice were intraperitoneally injected with D-Luciferin (150 mg/Kg) and major organs were harvested for imaging using IVIS (PerkinElmer Inc). For mmRNA expression kinetics of intratumorally-injected mmFluc LNPs, mice were imaged every 24 h as described above.

### mmRNA translation inhibition by mmPE LNPs

B16F10.9 were seeded at 3×10^5^ cells/well and cultured as described above. 24 h after seeding, cells were treated with mmPE LNPs and mmFluc LNPs as control at the indicated concentrations. 2 h post treatment, cells were transfected with 0.25 µg/mL EGFP mRNA (TriLink, USA) via messenger max transfection reagent (Invitrogen, USA) according to the manufacturers' recommendations. 12 h post EGFP transfection, cells were trypsinized, resuspended in PBS/1%FBS and 10^4^ cells were measured by flow cytometry (Cytoflex, Beckman Coulter, USA).

### *In-vivo* transfection of tumoral B16F10.9 cells by I.T. injection of LNPs

Eight to ten weeks-old female C57BL/6 mice (Envigo, Rehovot, Israel) were inoculated subcutaneously to the right flank with 10^5^ B16F10.9-mCherry-Luc labeled cells suspended in 50 µL HBSS. Ten days post inoculation, mice were I.T. injected with mmEGFP-LNPs (mRNA dose: 0.15 mg/Kg). 24 h post injection, tumors were dissected, and tumor single cells were extracted according to the manufacturer's recommendations (mouse tumor dissociation kit, Milteny, USA). 10^5^ cells were analyzed by flow cytometry (Cytoflex, Beckman Coulter, USA).

### Statistical analysis

Statistical analysis for comparing two experimental groups was performed using two-sided Student's t tests. Kaplan-Meier curves were used to analyze survival. A value of P < 0.05 was considered statistically significant. Analyses were performed with Prism 7 (GraphPad Software). Differences are labeled n.s. for not significant, * for P ≤ 0.05, ** for P ≤ 0.01, *** for P ≤ 0.001, and **** for P ≤ 0.0001. Pre-established criteria for the removal of animals from the experiment were based on animal health, behavior, and well-being as required by the ethical guidelines.

## Results

### Toxin-encoding mmRNA LNPs design and characterization

**Figure 1:**
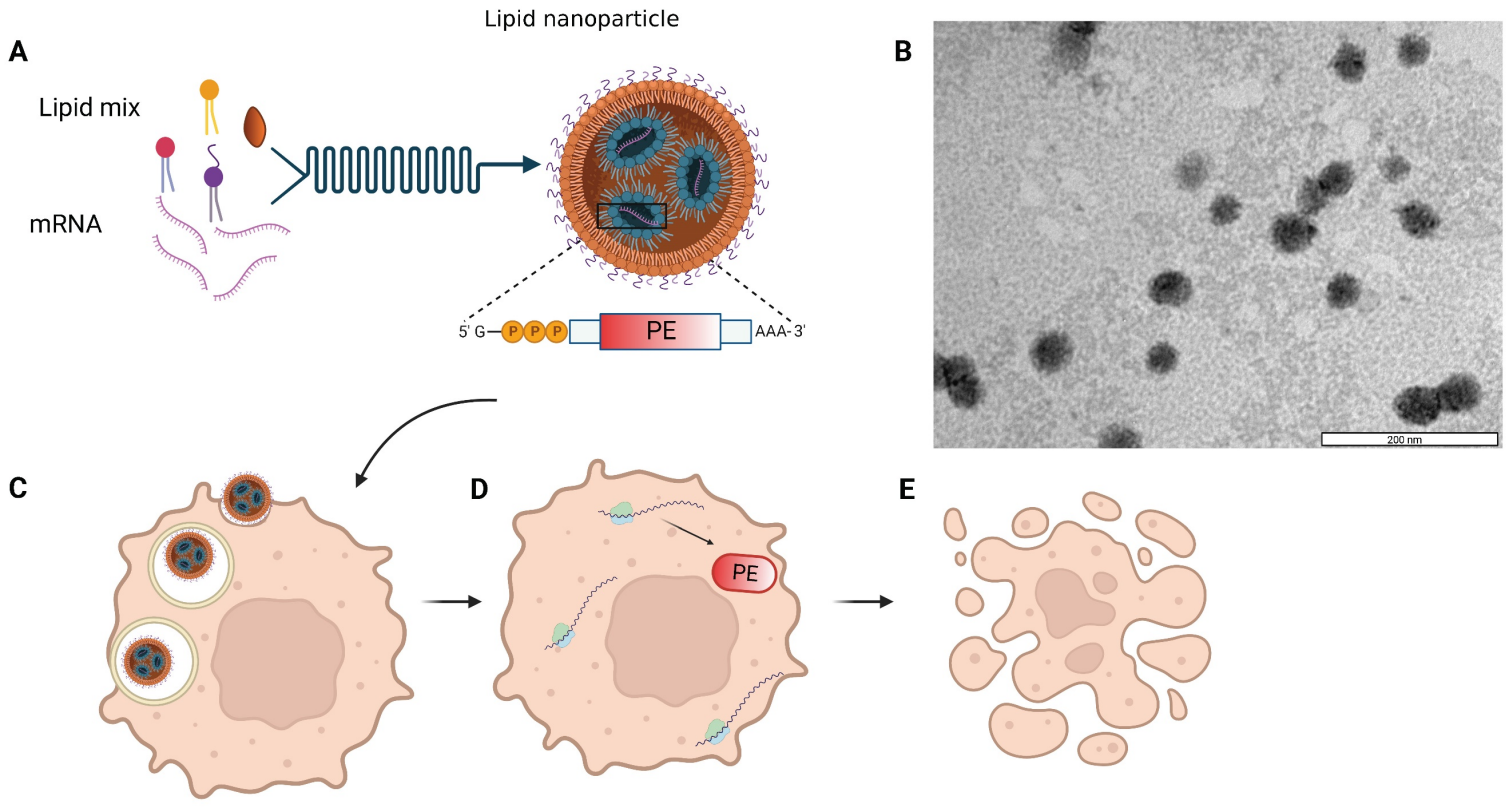
** Schematic and microscopic representation of toxin encoding mmRNA-loaded lipid nanoparticles.** A. Self-assembly of lipid mixture and mRNA molecules in acidic buffer composes mRNA-LNPs. mRNA is encoding for the pseudomonas exotoxin A (PE) toxin, which is delivered by the LNPs to cancer cells (C). The delivered mmPE is then translated by target cells into PE toxin (D) that induces apoptosis (E). B. Representative TEM image of Firefly Luciferase mmRNA-loaded LNPs. Bar scale - 200 nm.

We utilized *in-vitro* transcribed mRNA, into which chemically modified nucleotides were incorporated, shortly termed modified mRNA (mmRNA), encoding for either Firefly Luciferase, EGFP or PE, to optimize LNPs formulation. Modified bases incorporation into the *in-vitro* transcribed (IVT) mRNA is a well-investigated method to enhance protein expression and lower mRNA immunogenicity 7,37-38. LNPs were synthesized using the NanoAssemblr® microfluidic mixing system (Precision Nanosystems Inc., Vancouver, Canada), in which mmRNA molecules interact with ionizable lipids in acidic conditions and self-assembled with other components to form highly uniform nanoparticles (Figure 1). This controlled electrostatic interaction between the mRNA molecules and the ionizable lipids, allows an efficient encapsulation of the mRNA payload on the one hand, while avoiding the positive charge of the traditional cationic lipids, which is known to enhance toxicity 3,39-44. We characterized the size distribution and ζ potential of the mmRNA-LNPs using dynamic light scattering (DLS) measurements. To assess mmRNA encapsulation efficiency, we used the Quant-it™ Ribogreen assay as previously reported 36.

**Figure 2:**
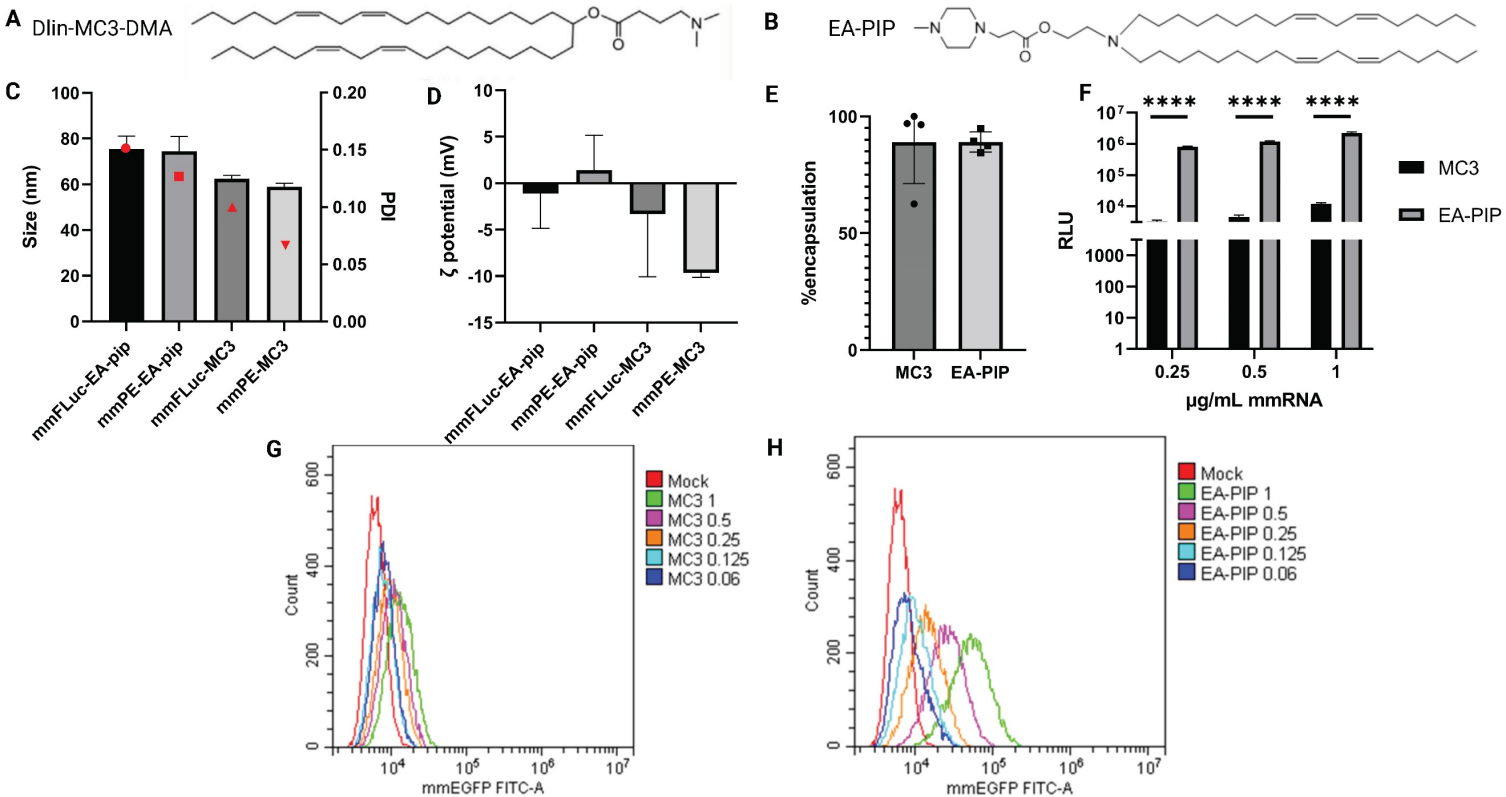
** Physicochemical characterization and *in-vitro* expression of MC3-mmRNA-LNPs and EA-PIP-mmRNA-LNPs.** A-B. Chemical structures of MC3 (A) and EA-PIP (B) ionizable lipids. C-D. LNPs' size and zeta potential measurements by dynamic light scattering (DLS). E. mmRNA encapsulation efficiency as reflected in a RiboGreen-based assay, allowing mmRNA concentration calculation, according to the absorbance of an RNA-binding dye. F. Firefly luciferase expression in B16F10.9 cells 48 h post incubation with mmFluc-LNPs composed of either MC3 or EA-PIP lipids. H-G. EGFP expression level in B16F10.9 cells 48 h post incubation with mmEGFP-LNPs composed of either MC3 (H) or EA-PIP (I) lipids. Multiple paired t-tests statistical analysis was performed by Prism GraphPad, *p ≤ 0.05, ****P < 0.0001.

To facilitate optimal mmRNA encapsulation in lipid nanoparticles, allowing both mmRNA protection and delivery while maintaining minimal toxicity, we encapsulated *in-vitro* transcribed mmRNA in LNPs composed of a novel ionizable lipid that was developed by our group, named EA-PIP 35,45. We compared EA-PIP (Figure 2B) with the clinically approved, well-studied ionizable lipid Dlin-MC3-DMA (MC3) (Figure 2A). We showed that while both EA-PIP LNPs and MC3 LNPs had an average size distribution below 100 nm and a similar encapsulation efficiency, they differed in their ζ potential, which was more consistent and closer to neutral in EA-PIP LNPs, while MC3 LNPs were marginally negative (Figure 2C-E).

To evaluate their functionality, we examined mmRNA-LNPs loaded with either Firefly Luciferase or EGFP mmRNA on different cancer cell lines, from both human and murine origin. Like MC3 LNPs, EA-PIP LNPs did not exhibit any significant cytotoxicity to cells when loaded with reporter mmRNA molecules (Figure S1). However, they demonstrated improved *in-vitro* expression of delivered mmRNA over MC3 LNPs, in agreement with our previous publication 46. We observed this dose-dependent effect for both mmFluc-LNPs and mmEGFP-LNPs, as reflected by increasing luminescence and fluorescence intensities, respectively (Figure 2F-H, S2, S7). In mmEGFP LNPs- treated cells, we could show that in high mmRNA concentrations (0.5 and 1 µg/ mL), there was only one population of cells expressing EGFP (Figure 2F-I).

### Pseudomonas exotoxin A mmRNA (mmPE) encapsulated in LNPs induces high rate of cancer cell apoptosis and attenuates protein translation *in-vitro*

**Figure 3:**
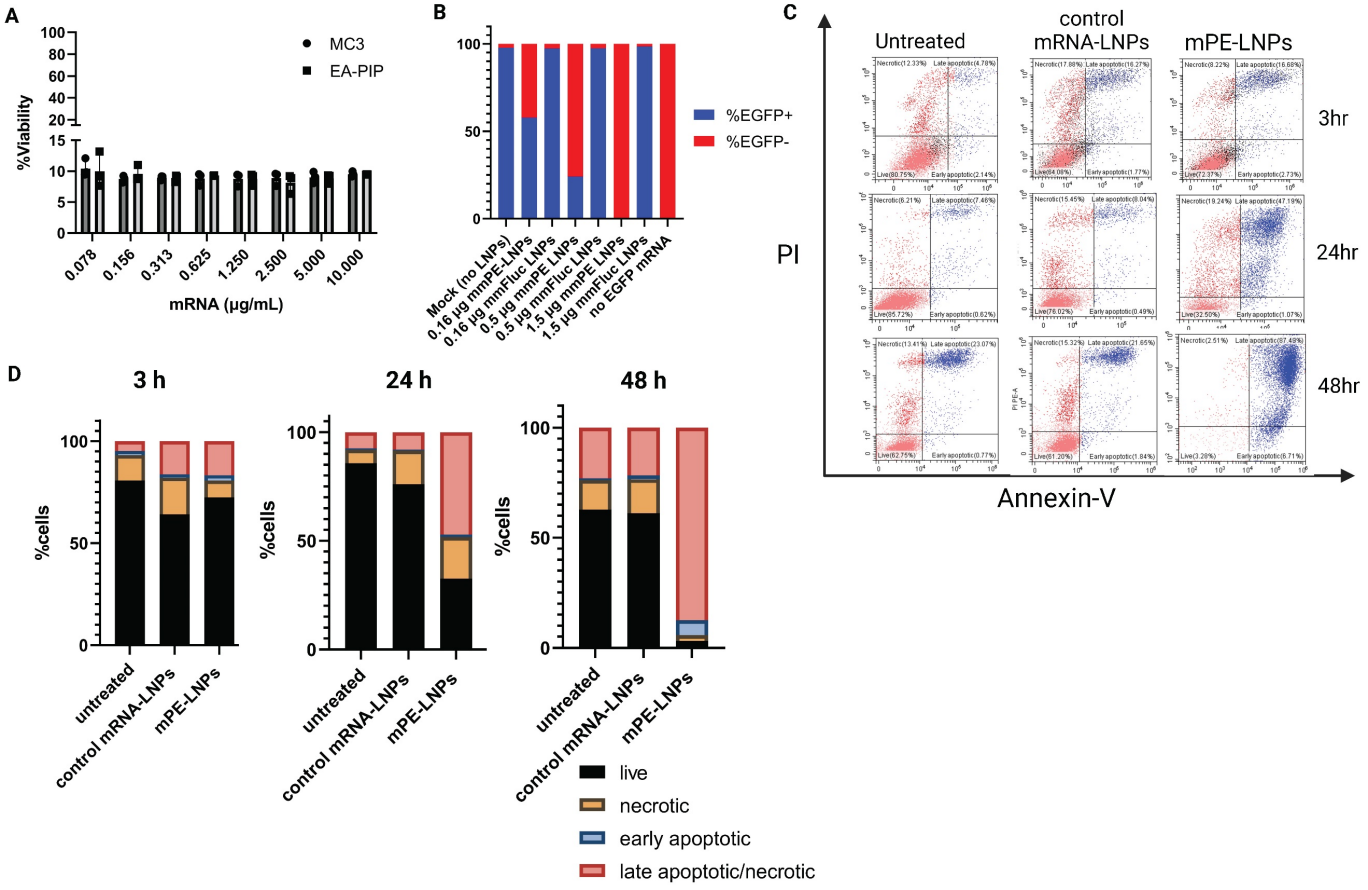
** mmPE-LNPs therapeutic effect *in-vitro*: cancer cell viability reduction via apoptosis and protein translation inhibition.** A. B16F10.9 cell viability rate 48 h post treatment with mmPE-LNPs. Cells were incubated with increasing doses of mmPE-LNPs, composed of either MC3 or EA-PIP lipids. 48 h post treatment, the viability rate of treated cells was exceptionally low (~10%) in all tested conditions. B. B16F10.9 cells pre-treated with mmPE-LNPs, had lower expression levels of transfected EGFP mmRNA compared to cells pre-treated with mmFluc LNPs, suggesting that mmPE LNPs inhibit protein translation. C. PI-Annexin assay for determination of necrosis and apoptosis rates 3 h, 24 h and 48 h post treatment. D. Graphical representation of PI-Annexin-V-stained cells according to their viability state: live, necrotic, early apoptotic or late apoptotic at all tested timepoints, indicating mmPE-LNPs caused significant apoptosis 48 h post treatment.

Next, we encapsulated mmRNA encoding for the pseudomonas exotoxin A domain III, which is the catalytic domain of the PE toxin, in LNPs. We hypothesized that encoding the active domain only, without the binding and translocation domains of the original toxin, will allow potent intracellular effect upon mRNA translation, while reducing the risk of side-off effects in case of toxin release from target cells. We assessed these mmPE LNPs' ability to induce cancer cell death *in-vitro*. We incubated B16F10.9 and other cancer cell lines with increasing amounts of encapsulated PE mmRNA (mmPE) and demonstrated a significant reduction in cancer cell viability 48 h post treatment (Figures 3A, S3). We also confirmed that this cytotoxicity was due to apoptosis, using a PI-Annexin-V staining and FACS analysis. B16F10.9 cells treated with mmPE-LNPs had an increasing fraction of apoptotic cells starting from 24 h post treatment to a massive rate of ~90% late-apoptotic cells 48 h post treatment (Figures 3C-D, S8).

To further validate that our mmPE-LNPs mechanism of action correlates the original PE toxin mechanism 24,28, and that the encoded mmPE triggers protein translation inhibition, we performed an *in-vitro* protein translation measurement, using a reporter gene mmRNA. We pre-incubated B16F10.9 melanoma cells with either mmPE LNPs or mmFluc LNPs for 2 h, and then transfected them with EGFP mmRNA using a commercial transfection reagent. We demonstrated EGFP expression inhibition in cells pre-treated with mmPE-LNPs compared to cells pre-treated with mmFluc LNPs and this effect was dose-dependent (Figures 3B, S6).

### Intratumorally administered mmPE-LNPs lead to intratumoral apoptosis and tumor growth inhibition in a B16-melanoma mouse model

To test mmPE-LNPs efficacy *in-vivo*, we intratumorally injected B16-melanoma tumor-bearing mice with either mmFluc-LNPs or mmPE-LNPs (0.15 mg/Kg) or PBS as a negative control (n = 6 mice / group). The treatment regimen included four intratumoral doses, with a gap of 2-3 days between injections and final analysis 72 h post the last injection (Figure 5A). These experiment settings and treatment protocol corresponds mmFluc LNPs expression kinetics upon intratumoral injection showed in a different experiment (Figure S4 D&E). Firefly luciferase expression caused high luminescence in the tumor 24 h post intratumoral administration, with a decay in the signal over time up to 96 h post administration (Figure S4).

Mice receiving mmPE-LNPs had smaller tumor volumes in all tested timepoints, and significantly lower tumor volumes at the experiment endpoint compared to the control group, as reflected by both tumor volumes measured in the whole animal, and *ex-vivo* tumor sizes (Figure 5C-D). We also did not observe a significant reduction in the average weight of the treated mice compared to the control mice, suggesting there was no substantial systemic toxicity of the treatment (Figure 5B).

**Figure 4:**
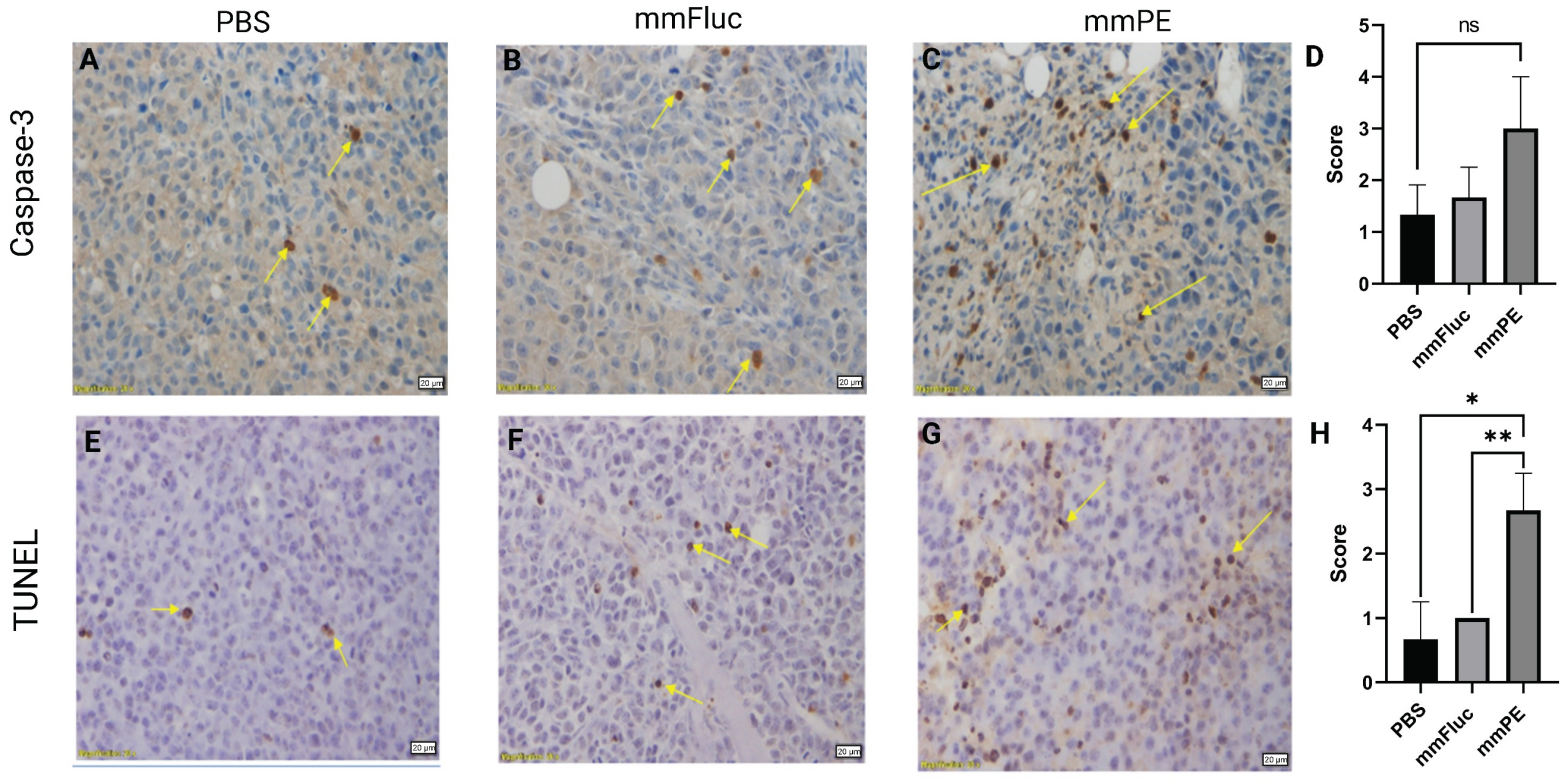
** Intratumorally-injected, repeated doses of mmPE-LNPs caused *in-vivo* apoptosis in tumor cells.** Representative images of mice tumors IHC stained for either caspase-3 (A-C) or TUNEL (E-G), yellow arrows mark stained cells. Left panel (A, E) represents PBS-treated mice, middle panel (B, E) represents mmFluc-LNPs treated mice and right panel (C, G) represents mmPE-LNPs treated mice (0.15 mg/Kg, 50 µl, four doses). All specimens (N = 3) were classified by a veterinary pathologist according to the following: Grade 0 = no positive reaction at all, Grade 1= Only few cells are positive (< 5 cells per a X20 field), Grade 2 = Very mild positive stain (5-15 cells per a X20 field), Grade 3 = Mild positive stain (15-25 cells per a X20 field), Grade 4= Moderate positive stain (25-50 cells per a X20 field), Grade 5 = Marked positive stain (> 50 cells per a X20 field). D&H are graphical representations of the average scoring of caspase-3 and TUNEL positive cells, respectively.

To strengthen these observations and validate the mechanism of action of the toxin encoded by the therapeutic mmRNA, PE, which is known to induce protein synthesis arrest leading to apoptosis 24,28, we harvested tumors from the mice at the final day of the experiment and analyzed them using immunohistochemistry staining. We employed caspase-3 staining to detect early apoptotic changes, and TUNEL assay to detect late apoptosis reflected by DNA fragmentation. We observed higher staining for both caspase-3 and TUNEL in the mmPE LNPs-treated group compared to the control groups, with a significantly higher scoring of TUNEL staining in mmPE LNPs group compared to the controls, indicating late apoptosis in the treated tumors at the experiment endpoint (Figure 4).

### Safety profile of intratumorally administered mmPE LNPs

Systemic toxicity and side-off effects are main concerns when developing a potent anti-cancer therapy. In addition to monitoring mice weight *in-vivo*, indicating there is no substantial adverse effect that caused weight loss, we also performed a biodistribution test and demonstrated minimal mmRNA expression in the main blood-filtrating organs upon mmFluc LNPs I.T. injection (Figure S4A-C).

We have evaluated general toxicity by liver and spleen histology, liver enzyme levels and pro-inflammatory cytokines measurement in the blood. H&E staining of liver and spleen tissues of mice receiving four doses of either PBS, mmFluc LNPs or mmPE LNPs (0.15 mg/Kg mmRNA) indicated that there were no significant changes in tested tissues compared to healthy mice, three days post last injection (Figure 6A-H). Additionally, a single, intratumoral dose of mmPE LNPs (0.5 mg/Kg) did not affect serum liver enzymes levels, as compared to untreated mice 24 h post administration (Figure 6I). Furthermore, we could not observe any detectable levels of either IL-6 or TNF-α in mice's serum 2 h and 24 h post mmPE LNPs intratumoral injection (data not shown).

### Repeated doses of mmPE-LNPs inhibited tumor growth and increased overall survival rate

Next, we aimed to evaluate the effect of our treatment on mice survival rate and determine tumor growth inhibition in a quantitative manner. For that, we utilized mCherry-Luciferase-labeled B16F10.9 cells for tumor inoculation. Mice were intratumorally injected with PBS, mmFluc LNPs or mmPE LNPs, every 2-3 days. Each mouse was treated until reaching an ethical sacrificing criterion of tumor volume of 1500 mm^3^, and then was sacrificed. Overall, the experiment lasted 35 days from tumor inoculation, with 10 injection timepoints (Figure 7A). The tumors' mCherry and Luciferase intensities were tracked using *in-vivo* imaging system (IVIS) every 2-3 days until the experiment endpoint. We could exhibit tumor growth inhibition, reflected in either tumor size measured with Caliper, mCherry signal and Luciferase signal in the mmPE-LNPs treated group compared to the control groups (Figures 7C&D, 8). Mice receiving mmPE-LNPs had reduced tumor volumes in all tested timepoints, and significantly lower tumor volumes at days 20, 22 and 25 post tumor inoculation compared to the control group (Figure 8B&D). We could also show a significant increase in the survival rate of mmPE-LNPs treated mice, compared to the control mice, with no considerable average weight loss (Figure 7B&E).

Facilitating the same mCherry-Luciferase-labeled tumor model, we were able to demonstrate cancer cells-specific transfection *in-vivo*. We displayed expression of mmEGFP delivered intratumorally by our LNPs. We observed that 44-60% of the mCherry-labeled cells expressed EGFP 24 h following a single mmEGFP LNPs intratumoral injection. Delivery specificity was excellent, as a great part of the EGFP-expressing cells *in-vivo* were mCherry positive, indicating most LNPs have reached cancer cells (Figures 7F, S5).

## Discussion

LNPs are rapidly emerging in recent years as vehicles for mRNA delivery and were shown to be a flexible and quickly scalable platform for this purpose 47-50. Their clinical application for inducing tumor cell death is emerging as a novel, promising field. The mainstream of such developing therapies nowadays are immunotherapies, including cancer vaccines employing mRNA encoding for tumor associated antigens 51-54, mRNA expressing monoclonal antibodies against such antigens 55, and immunomodulating cytokines such as IL-12 and TGF-β- encoding mRNA 56-59.

However, there is a need for alternative treatments in tumors that do not express an identified unique antigen, or tumors which are resistant or unresponsive to immunological treatments. Expressing a cytotoxic gene by the tumor cells represents a more straightforward approach, which could be useful in such indications. In this respect, mmRNA-entrapped in LNPs has the potential to expend the therapeutic index of the expressed lethal protein, as the toxic element is not found in the bloodstream, and it is produced at the site of mmRNA internalization.

To our knowledge, similar studies aiming to intratumorally express a toxin or a cytotoxic protein made use of either cationic lipid-based formulations, or a protamine-lipid formulations 60-62. While ionizable lipid- based lipid nanoparticles have close-to neutral surface charge, positively charged nanoparticles are known to have an increased *in-vivo* surface protein corona formation, accelerated elimination by the reticuloendothelial system (RES), non-specific uptake and high immunogenicity 3,39-44. The delivery of toxin-encoding mRNA entrapped in lipid nanoparticles is more clinically- relevant and preferable when immune stimulation is unnecessary.

In addition, we suggest LNP-mediated mmRNA delivery to tumor cells as an alternative to immunotoxins, which require receptor binding, intracellular processing and trafficking for functionality. Tumor cells can alter these processes to acquire resistance to the treatment 27,33. Like previously stated regarding mRNA-based vaccines, immunotoxins provide targeting to cancers expressing known cell-surface antigens, and so are irrelevant for other types of cancer. Moreover, there is no clinically- approved immunotoxin-based therapy for solid tumors indications to date. This may be due to low distribution and penetration of large molecules into solid tumors 27.

Furthermore, receptor shedding from the cellular membrane can hamper immunotoxins internalization. For instance, a mesothelin-targeted immunotoxin currently under clinical evaluation showed differentiated effect on tumors originated from different mesothelin expressing cell lines, and the researchers claimed that the target receptor shedding might be the reason 23. Low receptor expression on target cells can also decrease efficacy, as seen for the immunotoxin BL22 that was tested in CLL patients 32. Some immunotoxins can still bind normal cells and lead to vascular leak syndrome, in which endothelial cells are damaged, so fluid from circulation leaks into the tissues and can lead to edema, a fall in serum proteins, hypotension, and vascular collapse 20. In addition, mAb conjugation may reduce the anti-cancerous toxic effect of the toxin. The conjugation of F1G4 mAb to Abrin toxin, for example, lowered the cancer cell death rate *in-vitro,* compared to the free toxin 21. We assume that intracellular synthesis of the intact, catalytically-active toxic domain, can overcome such limitations. mmRNA delivery into tumor cells allows such intracellular synthesis, due to the universality of the genetic code of mRNA molecules. In an interesting study that might address some of those challenges, the investigators showed that immunotoxin expression and secretion by human primary T cells transfected with immunotoxin-encoding mRNA, caused cancer cell death when co-cultured with ovarian cancer cells. However, the cytotoxic effect was mild and *in-vivo* evaluation was not presented 63.

**Figure 5:**
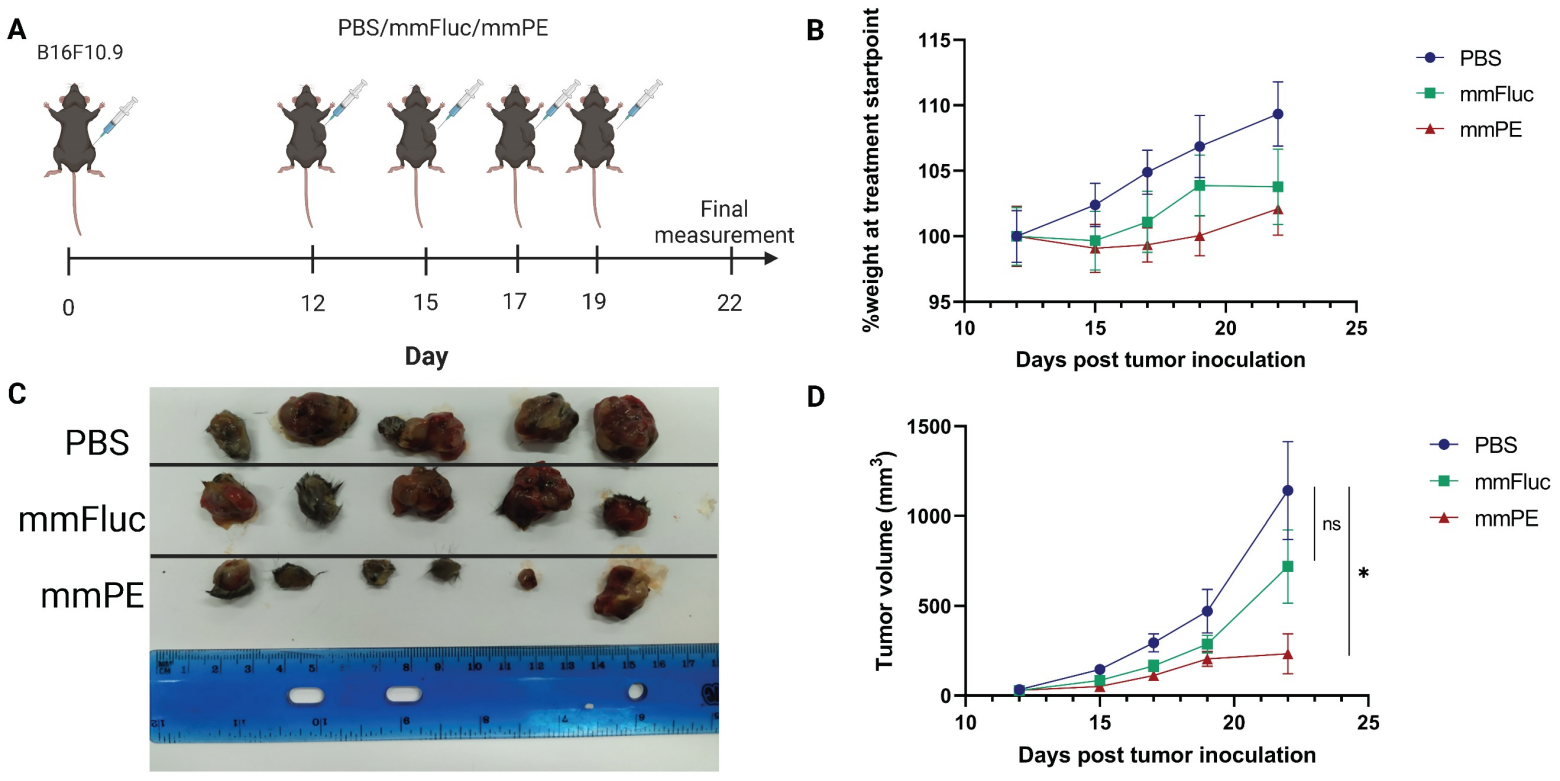
** Intratumorally-injected mmPE-LNPs effect on tumor growth in a B16F10.9 mouse model.** A. Experiment settings and timeline. Mice were subcutaneously inoculated with B16F10.9 melanoma cells and were treated with four intratumoral, repeated doses of either PBS, mmFluc LNPs or mmPE LNPs (0.15 mg/Kg, 50 µl), starting 12 days post tumor inoculation. B. Average percentage of mice's weight compared to first day of treatment, representing weight loss rate. C. *Ex-vivo* tumors at experiment endpoint, visually demonstrating tumor sizes. D. Average tumor volume of mice from first day of treatments [n = 6 mice / group. Student's t-test statistical analysis of every pair of groups was performed using Prism GraphPad, *p ≤0.05.

**Figure 6:**
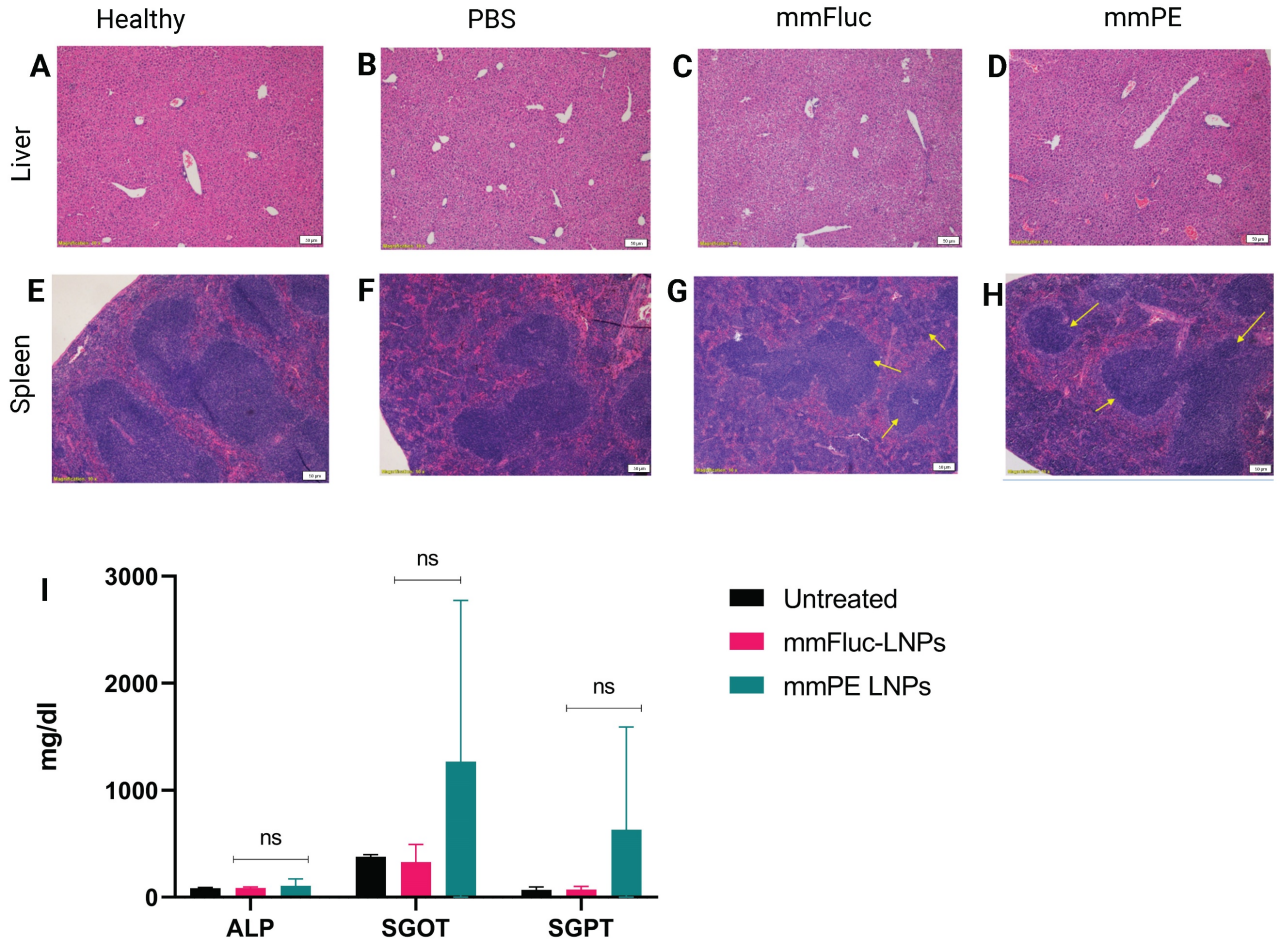
** Safety profile analysis of mmPE-LNPs.** A-H. Histology of liver (A-D) and spleen (E-H) samples (hematoxylin and eosin staining, representative images) of either healthy (A,E), PBS-injected (B,F), mmFluc-LNPs-injected (C,G) or mmPE-LNPs-injected mice (D,H), after four intratumoral injections, 0.15 mg/Kg, 50 µl. I. Liver enzymes of mice intratumorally injected with either mmFluc LNPs or mmPE LNPs, compared to untreated mice, 24 h post injection (0.15 mg/Kg, 50 µl), indicating no significant increase has occurred in the treatment group.

**Figure 7:**
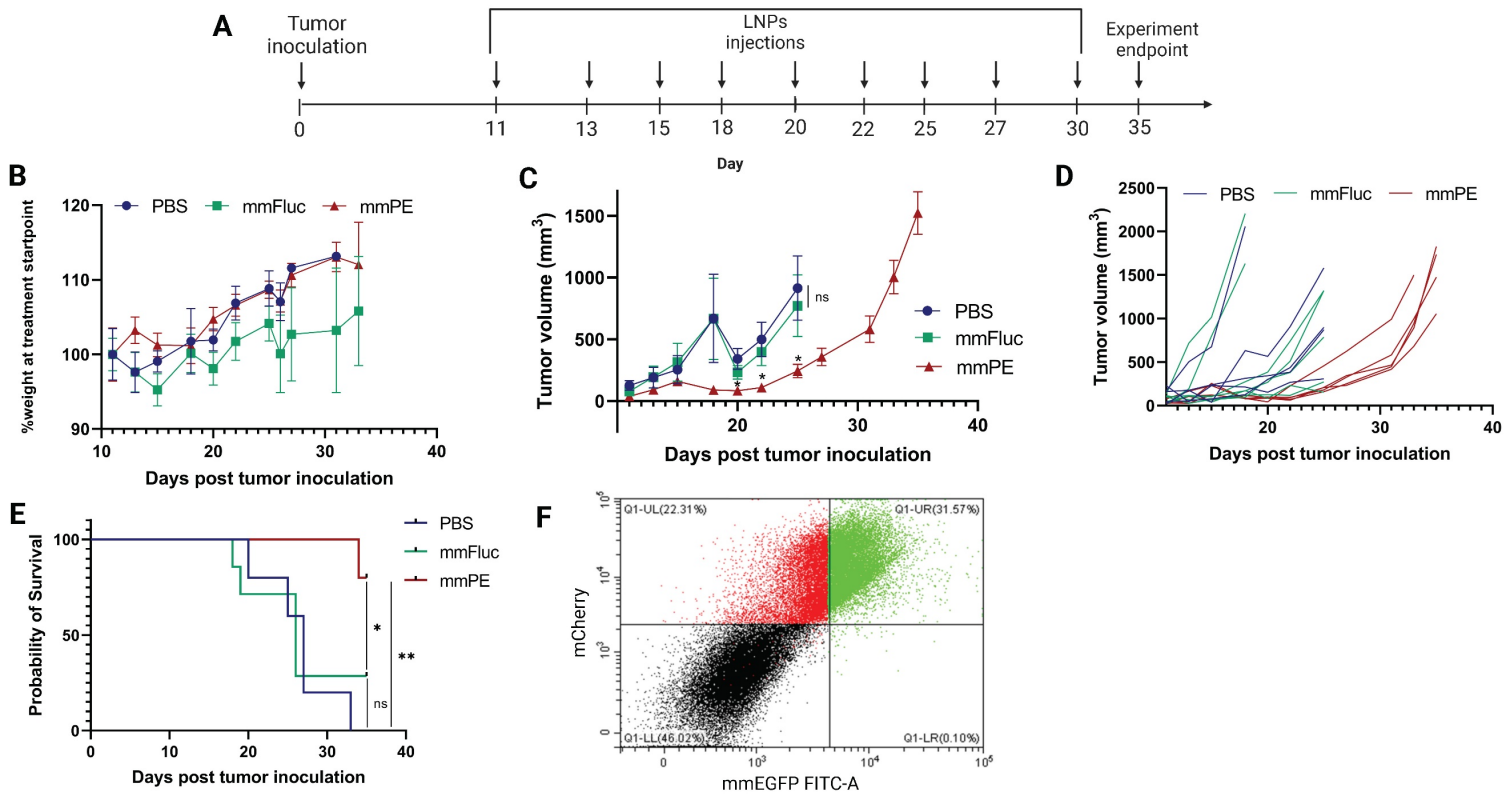
** Intratumorally injected mmPE-LNPs caused tumor volume reduction, cancer-cell specific expression and better survival rate, with minimal systemic toxicity.** A. Experiment timeline and settings. B. Average percentage of mice's weight compared to first day of treatment, representing weight loss rate. No significant weight loss in the treated group (mmPE, repeated doses, 0.15 mg/Kg, 50 µl) compared to the control groups (PBS and mmFluc) was observed. C. Average tumor volume of all groups. [n = 6 mice / group. Statistical analysis was done using student's t-test for every pair of groups, performed by Prism GraphPad, *p ≤0.05]. D. Tumor volume of each individual mouse until reaching sacrificing criteria (tumor volume ≥ 1500 mm^3^). E. Kaplan-Meier plot representing survival rate of all groups., showing significant positive effect of mmPE-LNPs on mice's survival rate. [Log-rank (Mantel-Cox) test was used for curve comparison using Prism GraphPad, *p ≤0.05, **p ≤0.01.]. F. Representative FACS analysis of mCherry-labeled tumor cells of mice receiving a single IT dose of mmEGFP-LNPs, 24 h post injection. A substantial fraction of almost 60% out of the mCherry-labeled cells also co-expressed EGFP (FITC), demonstrating a high delivery rate. Additionally, nearly all EGFP expressing cells were also mCherry positive, representing extremely high specificity.

Here we demonstrated the concept of utilizing mmRNA encapsulated in LNPs, as a feasible type of suicide-gene therapy, for the treatment of solid tumors. We showed that mmRNA encoding for the pseudomonas exotoxin A domain III encapsulated in LNPs, caused a significant anti-tumoral effect, both *in-vitro* and *in-vivo*. This treatment not only inhibited tumor growth, but also enhanced the overall survival of the treated animals. Furthermore, we could show that cancer cell death was due to apoptosis (Figures 3, 4). We also demonstrated that the molecular basis for cell apoptosis corresponds the known mechanism of action of pseudomonas exotoxin A (Figures 4B, S6). Additionally, although its high efficacy, our treatment did not cause any significant systemic toxicity, as reflected by animal weight, H&E staining of the liver and spleen sections, liver enzymes levels and the major cytokines evaluated (Figures 5B, 6, 7B). Moreover, tumor single cell analysis revealed high efficacy of our treatment, as a single I.T. dose resulted in 44-60% transfection of cancer cells (mCherry positive). Specificity was also high as negligible percent of EGFP-expressing cells were mCherry negative, meaning that the vast majority of the detected EGFP signal was expressed by mCherry-labeled-cancer cells (Figures 7F, S5).

**Figure 8:**
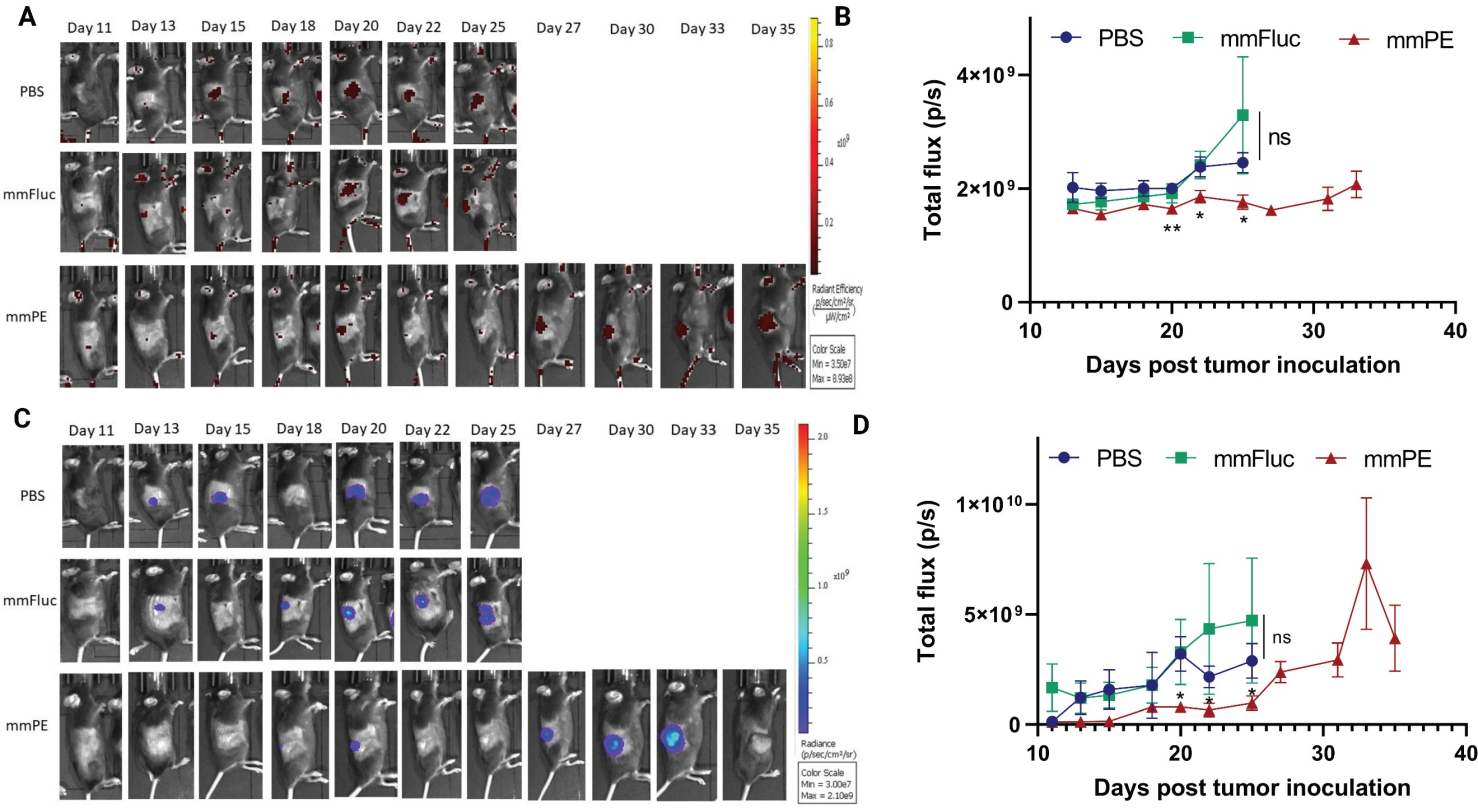
** Intratumorally-injected mmPE-LNPs inhibited B16-melanoma growth, as reflected by lower luminescence and fluorescence signals of labelled tumors.** A&C. Representative IVIS images showing mCherry-luc labelled B16F10.9 tumor progression as reflected by either mCherry (A) or Firefly-luciferase (C) signals starting 48 h post the first injection (PBS, mmFluc LNPs or mmPE LNPs, 0.15 mg/Kg, 50 µl). Mice reaching a threshold tumor size of 1500 mm^3^ were sacrificed and therefore had no imaging in later timepoints. Mice have reached larger tumors sooner in the control groups compared to the mmPE-LNPs treated groups. B&D. Average flux of mCherry (B) or Firefly Luciferase (D) signals from tumors of all groups over time showing that tumor signals were significantly lower in the treatment group for both Firefly luciferase and mCherry reporters at days 20, 22 and 25 post tumor inoculation, compared to the control groups. [Statistical analysis was done using student's t-test for every pair of groups, performed by Prism GraphPad, *p ≤0.05.]

One important drawback reported is the time limitation of the therapeutic window effect, as we observed a tumor relapse in late stages of the repeated dosing experiment (Figures 7C, 8). One explanation is that B16 melanoma models are known to be overly aggressive with a very rapid growth rate. Therefore, intratumoral injections have an inherent barrier in reaching massive portion of the tumor cells, in late stages of tumor growth. We cannot rule out antibody production against the PE toxin as the reason for tumor relapse. However, this explanation is less feasible due to the immunosuppressive activity of the PE toxin 64. Developing technologies for inducing targeted, tumor-specific mRNA expression upon systemic administration has the potential to overcome this obstacle, as the mmRNA-LNPs will reach the tumor more homogenously via blood circulation. We hope to further investigate into this aspect and demonstrate improved therapeutic effect in more tumor models in the future.

In conclusion, using toxin-encoding mmRNA entrapped in LNPs, can overcome some of the above discussed limitations of the current immunotoxin-based anti-cancer therapies, with an additional advantage of a safer payload delivery to tumor cells. This platform may represent a new class of treatment that can address an unmet clinical need for solid tumors therapy.

## Supplementary Material

Supplementary figures.

## Abbreviations

MC3: DLin-MC3-DMA
LNPs: Lipid nanoparticles
mmRNA: modified mRNA
PI: propidium iodide
FACS: Fluorescence-activated cell sorting
TEM: transmission electron microscopy
PE: pseudomonas exotoxin
mAB: monoclonal antibody
I.T.: intratumorally
