# Supplementary material for: Lipid nanoparticles-loaded with toxin mRNA represents a new strategy for the treatment of solid tumors
Source: Theranostics. 2023 Jun 12;13(11):3497–508. doi: 10.7150/thno.82228 (PMC10334842; doi:10.7150/thno.82228)
Supplement: Supplementary file 1 — Supplementary figures. [file thnov13p3497s1.pdf]

Lipid nanoparticles-loaded with toxin mRNA represents a new strategy for the treatment of solid tumors.

Yasmin Granot-Matok[1-4], Assaf Ezra[1-4], Srinivas Ramishetti[1-4], Preeti Sharma[1-4], Gonna Somu Naidu[1-4], Itai Benhar[2,4,5] and Dan Peer[1-4,*]

[1]Laboratory of Precision NanoMedicine, Shmunis School for Biomedicine and Cancer Research, George S. Wise Faculty of Life Sciences, Tel Aviv University, Tel Aviv 69978, Israel.
[2]Center for Nanoscience and Nanotechnology, Tel Aviv University, Tel Aviv 69978, Israel.
[3]Department of Materials Sciences and Engineering, Iby and Aladar Fleischman Faculty of Engineering, Tel Aviv University, Tel Aviv 69978, Israel.
[4]Cancer Biology Research Center, Tel Aviv University, Tel Aviv 69978, Israel.
[5]Laboratory of Antibody Engineering, Shmunis School for Biomedicine and Cancer Research, George S. Wise Faculty of Life Sciences, Tel Aviv University, Tel Aviv 69978, Israel.

* Corresponding author. Email: peer@tauex.tau.ac.il

Content

**Items**	**Pages**
Modified mRNA coding sequence of PE	2
Additional *in-vitro* expression of mmFluc-LNPs	2
in-vitro toxicity of reporter mmRNA-loaded LNPs	3
Additional *in-vitro* effect of mmPE-LNPs	3
in-vivo biodistribution and mmRNA expression kinetics of intratumorally-injected mmFluc LNPs	4
in-vivo co-localization of tumoral mCherry label and injected mmEGFP delivered by intratumoral injection of mmEGFP LNPs	5
EGFP mmRNA translation inhibition caused by pre-incubation with mmPE LNPs	5-6
Gating strategy for EGFP mmRNA expression analysis	6
Gating strategy for PI-Annexin assay	6

PE domain III cDNA sequence used for IVT mmRNA synthesis:

ATGgccgaagaagctttcctcggcgacggcggcgacgtcagcttcagcacccgcggcacgcagaactggacggtgg
agcggctgctccaggcgcaccgccaactggaggagcgcggctatgtgttcgtcggctaccacggcaccttcctcgaagc
ggcgcaaagcatcgtcttcggcggggtgcgcgcgcgcagccaggacctcgacgcgatctggcgcggtttctatatcgcc
ggcgatccggcgctggcctacggctacgcccaggaccaggaacccgacgcacgcggccggatccgcaacggtgccct
gctgcgggtctatgtgccgcgctcgagcctgccgggcttctaccgcaccagcctgaccctggccgcgccggaggcggc
gggcgaggtcgaacggctgatcggccatccgctgccgctgcgcctggacgccatcaccggccccgaggaggaaggcg
ggcgcctggagaccattctcggctggccgctggccgagcgcaccgtggtgattccctcggcgatccccaccgacccgcg
caacgtcggcggcgacctcgacccgtccagcatccccgacaaggaacaggcgatcagcgccctgccggactacgcca
gccagcccggcaaaccgccgcgcgaggacctgaagTAA

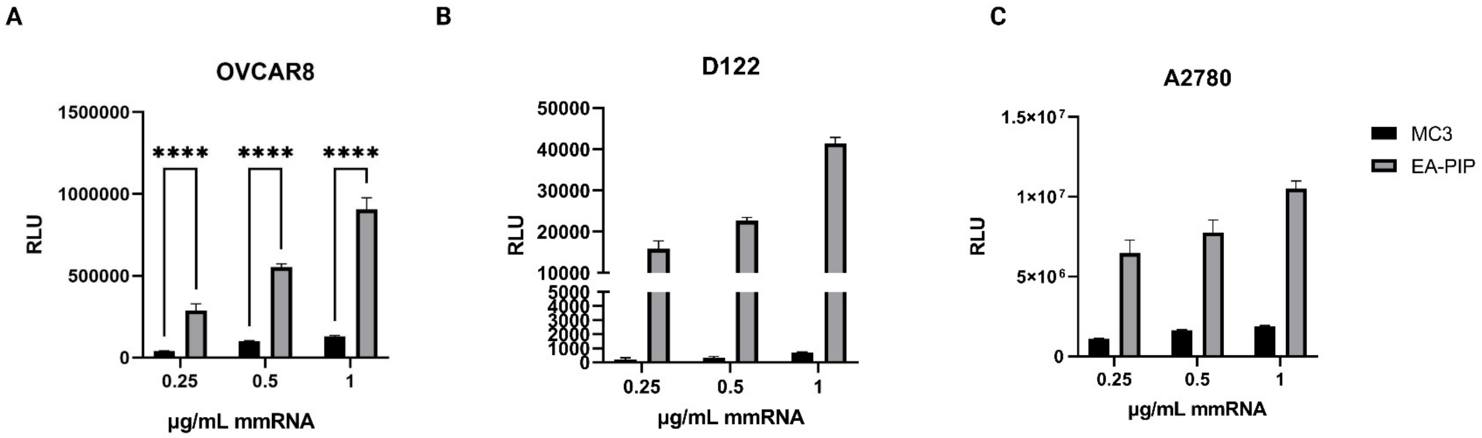

Figure S1. Additional *in-vitro* mmRNA expression comparing MC3 and EA-PIP in different cancer cell lines. OVCAR8 human ovarian cancer cell line (A) D122 Murine Lewis Lung carcinoma cell line (B) and A2780 human ovarian cancer cell line (C) were treated with increasing amounts of mmFluc-LNPs composed of either MC3 or EA-PIP. Cells were analyzed for luciferase expression 48 h post LNPs incubation. Average relative luminescent units (RLU) are presented for each tested concentration.

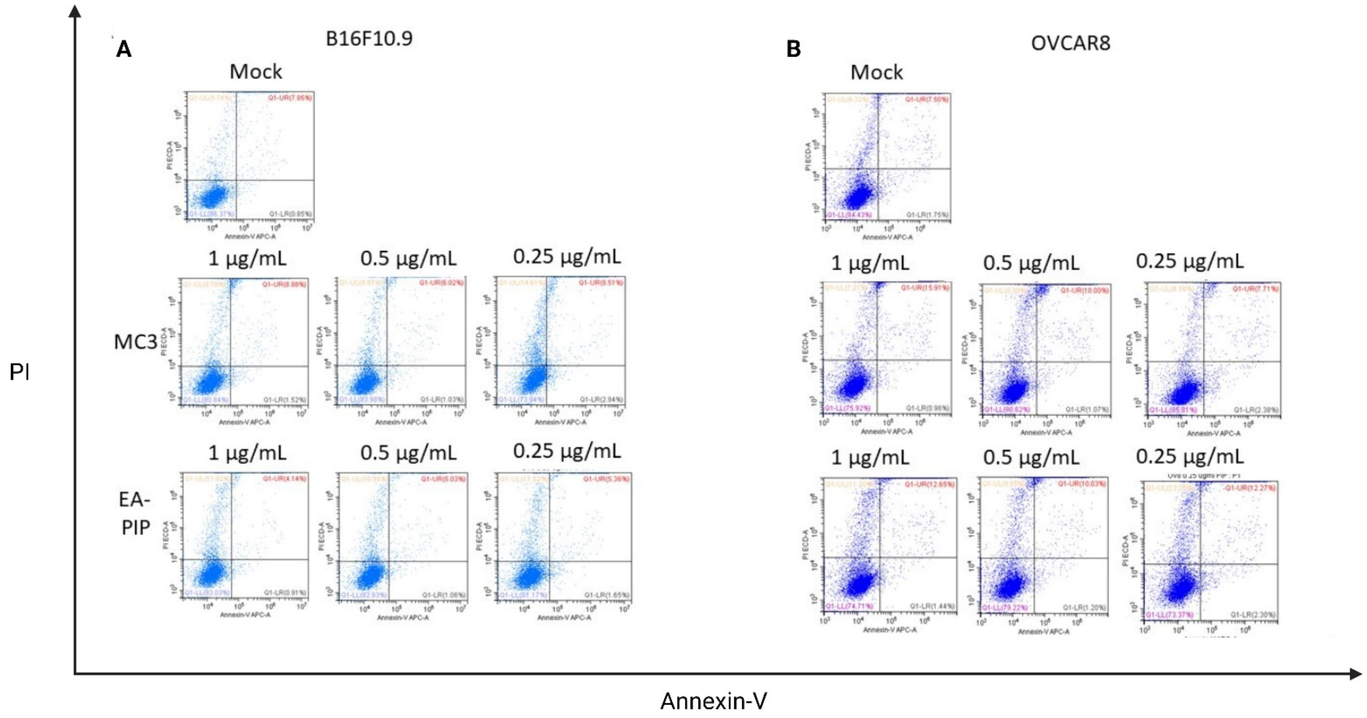

Figure S2. ***in-vitro*** **effect of reporter mmRNA-loaded LNPs on cancer cell viability.** B16F10.9 (A) murine melanoma cancer cell line and OVCAR8 (B) human ovarian cancer cell line were treated with increasing amounts of mmFluc-LNPs composed of either MC3 or EA-PIP. Cells were analyzed for apoptosis and necrosis rates using PI-Annexin-V assay 48 h post LNPs incubation.

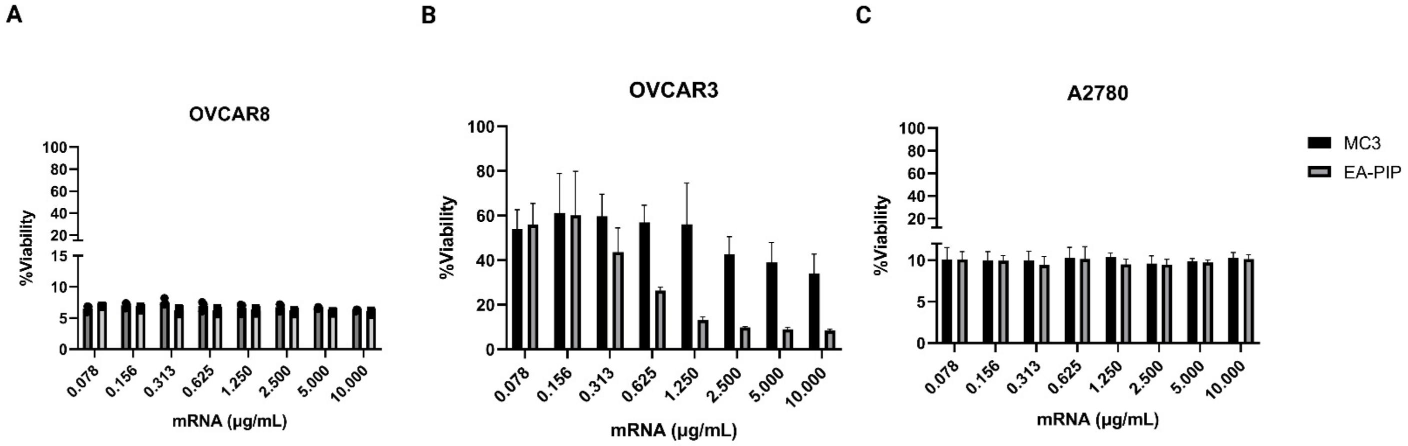

Figure S3. Additional ***in-vitro*** **effect of mmPE-LNPs on different cancer cell lines.** OVCAR8 (A) OVCAR3 (B) and A2780 (C) ovarian cancer cell lines viability 48 h post incubation with mmPE-LNPs composed of either MC3 or EA-PIP.

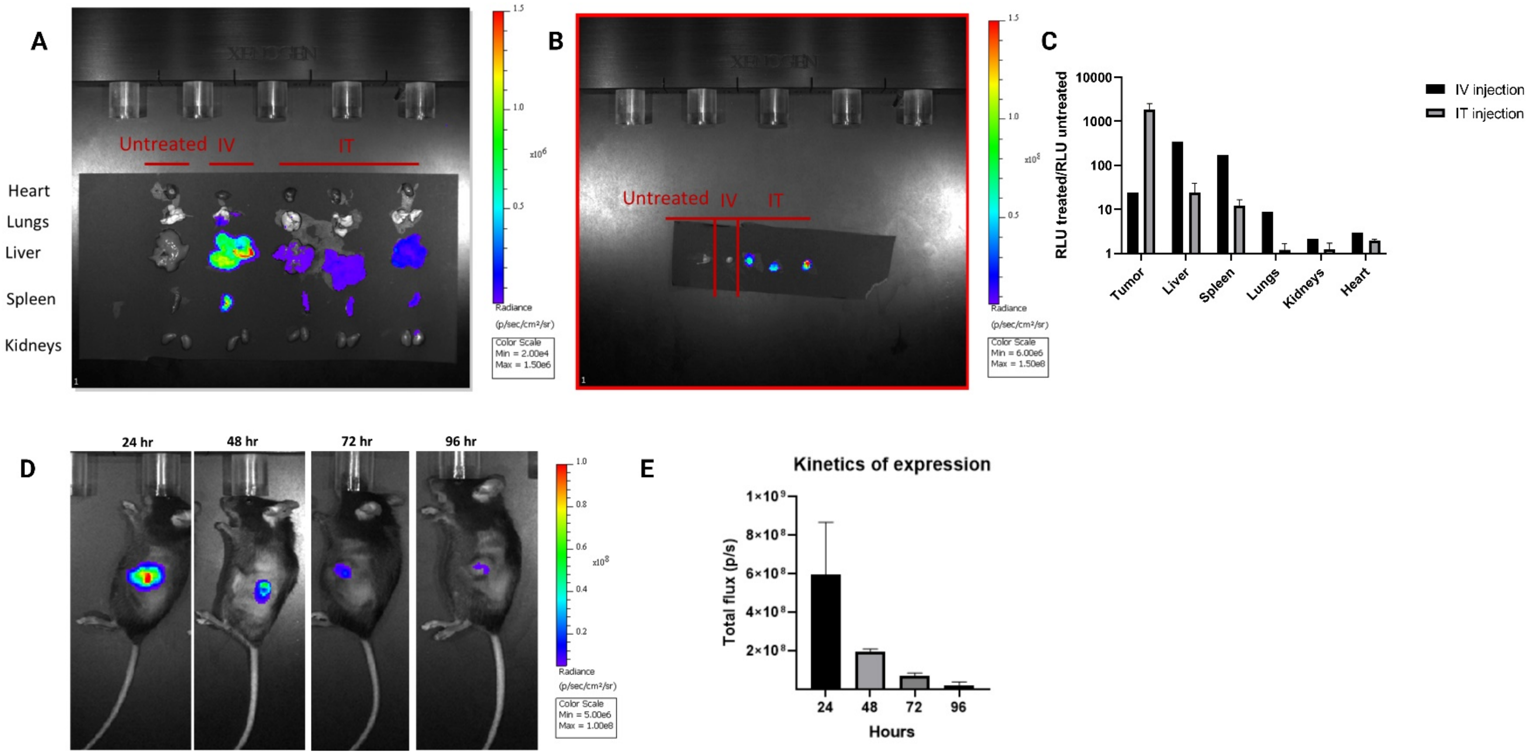

Figure S4. *In-vivo* biodistribution and mmRNA expression kinetics of intratumorally-injected mmFluc LNPs. A-C. Biodistribution of mmFluc-LNPs expression intratumorally administered to B16F10.9-tumor bearing mice (0.15 mg/Kg), 24 h post injection, as compared to untreated and intravenously injected mice. A. tumors of mice from all groups. B. main filtrating organs from all groups. C. Fold increase in luciferase signal (RLU) in intratumoral-injected mice as compared to intravenously injected mouse, in the tumor and different organs. D-E. *in-vivo* luciferase expression in B16F10.9 tumors 24 h, 48 h, 72 h and 96 h post single I.T. injection of mmFluc LNPs (0.15 mg/Kg), as reflected by IVIS imaging (D) and luminescent signal quantification (E).

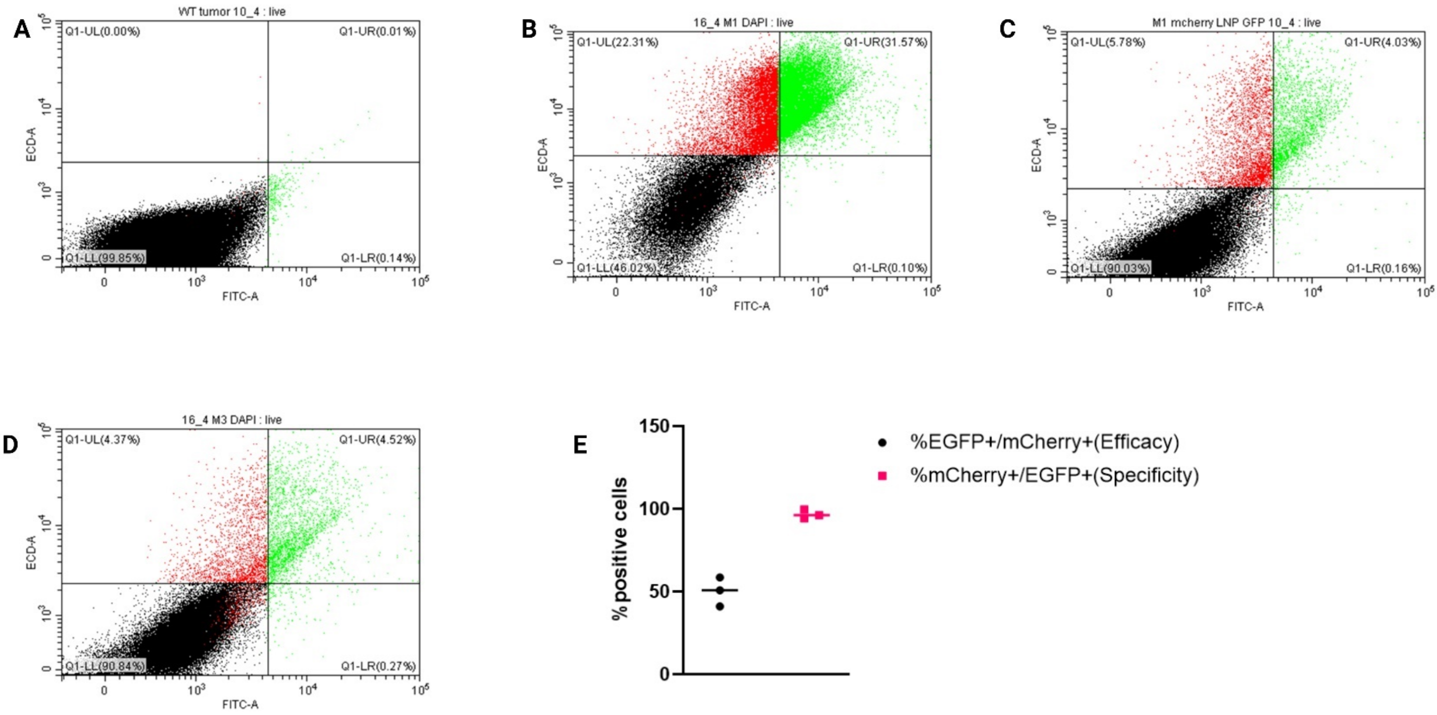

Figure S5. ***In-vivo*** **co-localization of tumoral mCherry label and mmEGFP delivered by intratumoral injection of mmEGFP LNPs.** A-D. FACS analysis of each of the treated mice tumors, representing EGFP expression (FITC) and mCherry (ECD). E. 24 h post intratumoral injection of mmEGFP LNPs, 44-60% of the mCherry-expressing tumor cells expressed the delivered mmEGFP. The EGFP expression specificity is represented as the mCherry positive cells percentage out of total EGFP expressing cells, which was almost 100%.

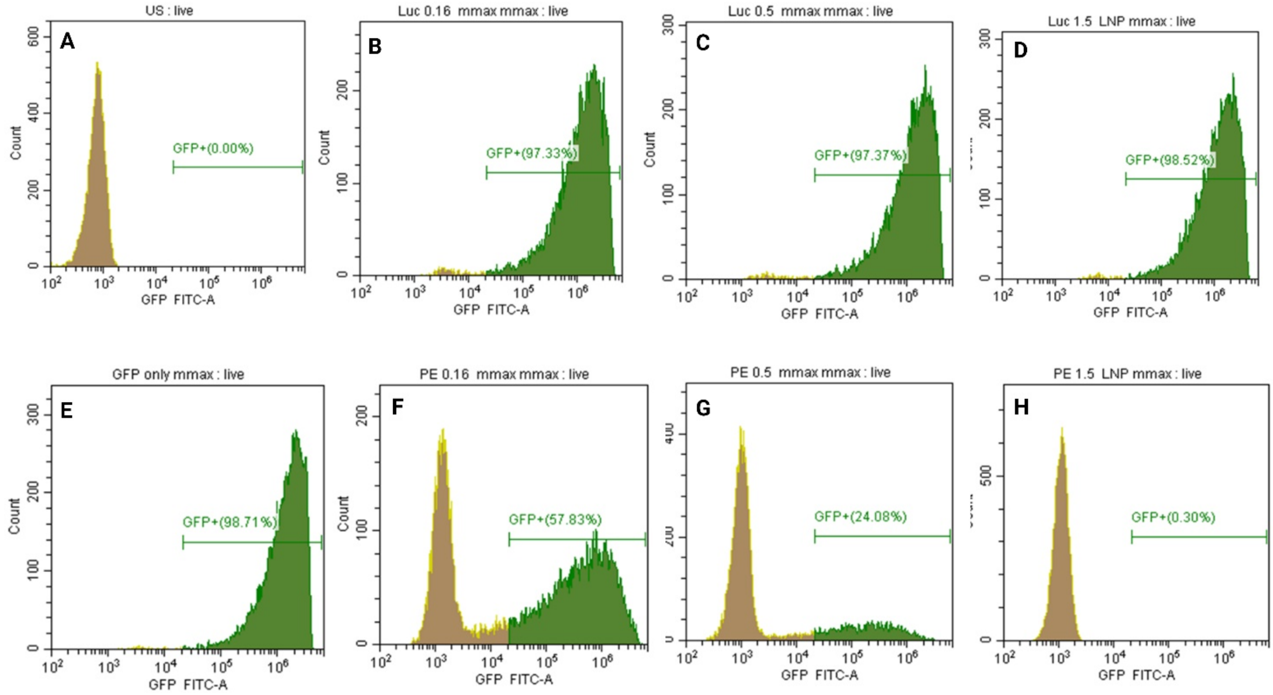

Figure S6. EGFP mmRNA translation inhibition caused by pre-incubation with mmPE LNPs. B16F10.9 cells were incubated overnight with either mmPE LNPs or mmFluc LNPs and then transfected with EGFP mmRNA using lipofectamine MessengerMax transfection reagent. Cells were analyzed by FACS 4 h post EGFP mmRNA transfection. A. Negative control: no pre-treatment and no EGFP mmRNA transfection. B-D. Cells pre-treated with increasing amounts of mmFluc LNPs: 0.16 µg (B), 0.5 µg (C) and 1.5 µg (D) and then transfected with EGFP mmRNA (0.25 µg/mL). E. Cells transfected with EGFP mmRNA (0.25 µg/mL) with no pre-treatment. F-H. Cells pre-treated with increasing amounts of mmPE LNPs: 0.16 µg (F), 0.5 µg (G) and 1.5 µg (H) and then transfected with EGFP mmRNA (0.25 µg/mL).

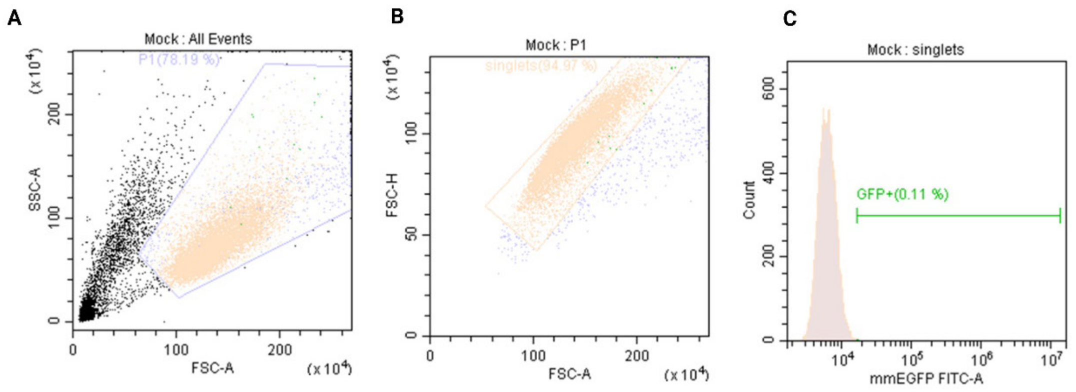

Figure S7. Gating strategy for EGFP expression analysis using FACS.

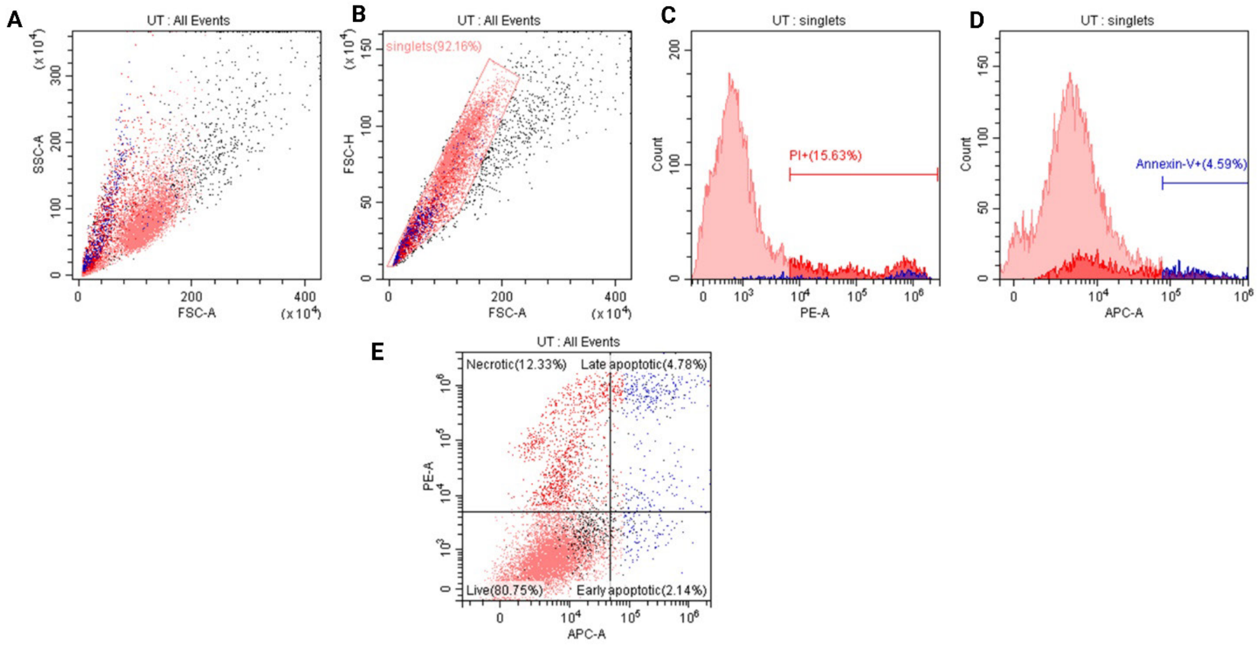

Figure S8. Gating strategy for apoptosis and necrosis analysis by PI-Annexin-V assay.
